# Field Measurement and Analysis on the Mechanical Response of Asphalt Pavement Using Large-Particle-Size Crushed Stone Base Treated with Fly Ash and Slag Powder

**DOI:** 10.3390/ma16237277

**Published:** 2023-11-22

**Authors:** Ruikang Yang, Xinzhong Gan, Liping Liu, Lijun Sun, Jiang Yuan

**Affiliations:** 1 The Key Laboratory of Road and Traffic Engineering, Ministry of Education, Tongji University, Shanghai 201800, China; rkyang@tongji.edu.cn (R.Y.); ljsun@tongji.edu.cn (L.S.); jyuan@tongji.edu.cn (J.Y.); 2 Yichun Highway Development Center, Yichun 336000, China; xinzhong_gan@163.com

**Keywords:** asphalt pavement, large-particle-size crushed stone base, fly ash and slag power, mechanical response, analysis of variance

## Abstract

The mechanical response of asphalt pavement under vehicular loading is an essential reference for crack-resistant pavement design. However, limited research focuses on the mechanical response measurement of asphalt pavement using a large-particle-size crushed stone base treated with fly ash and slag powder. Therefore, two types of asphalt pavements were constructed. The first type of asphalt pavement uses a large-particle-size crushed stone base treated with fly ash and slag powder, where the slag powder uses granulated blast furnace slag powder. The second type uses a conventional cement-stabilized crushed stone base and serves as a reference structure. Firstly, the strain gauges and temperature sensors were installed during the construction of asphalt pavements. Secondly, the mechanical response of the pavement was tested at different speeds and service time conditions. Then, sensitivity analysis and three-factor analysis of variance (ANOVA) were carried out. Finally, the prediction equations were developed. The results show that the longitudinal strain pulse of the asphalt layer exhibited a “compression–tension–compression” characteristic. For the transverse strain pulse of the asphalt layer, the base layer’s transverse and longitudinal strain pulses were only shown as “tensile” characteristics. The vehicular speed significantly affected the strain values for the base and asphalt layers, showing a decreasing trend with increasing speed. For the asphalt layer, the strain values showed an increasing trend with increasing temperature; for the base layer, the strain values showed a decreasing trend with increasing service time. The type of base layer had a significant effect on the strain value. Compared with the conventional base layer, the large-particle-size crushed stone base treated with fly ash and slag powder had lower strain at the base layer and a lower position of the asphalt layer, which could better prevent bottom-up fatigue cracking. Finally, the strain prediction model of the pavement under the speed and temperature (service time) was fitted to obtain a model that can predict the mechanical response of the pavement under different operating conditions. The findings of this research can provide a reference for the design of asphalt pavement using a large-particle-size crushed stone base treated with fly ash and slag powder.

## 1. Introduction

Asphalt pavement with a cement-stabilized crushed stone base is one of the most commonly used highway pavement structures [1,2,3]. This type of asphalt pavement has the advantages of high strength, high stiffness, and easy construction. However, due to the materials’ characteristics, the base layer is prone to cracks extending to the asphalt layer [4,5]. Moreover, owing to the rigidity of the cement-stabilized crushed stone base, the cost of maintaining the layer when it produces cracks is relatively high [6].

Based on the above problems, large-particle-size crushed stone materials were used in a base layer [7,8,9]. In addition, fly ash and slag powder were used to replace cement as a binder in the base layer to save energy and protect the environment [10]. Then, large-particle-size crushed stone materials treated with fly ash and slag powder were applied to the base layer [10]. To accurately design this type of asphalt pavement, it is necessary to know its mechanical response under vehicular loading. Therefore, the input parameter modulus required for calculating the mechanical response was obtained from laboratory tests for both the base and the asphalt layer. The stress and strain conditions of the specimen in the laboratory tests were quite distinct from those in the pavement [11,12]. Therefore, mechanical response measurement research was needed to more accurately grasp the mechanical properties of the asphalt pavement using a large-particle-size crushed stone base treated with fly ash and slag powder.

Relevant studies were investigated to obtain the strain and stress inside asphalt pavements [1,13,14,15,16,17,18,19,20,21]. Chatti et al. [13] measured transverse and longitudinal strains at the bottom of HMA layers at the PACCAR Technical Center in Washington, D.C. The material used for the base layer was graded gravel. Al-Qadi et al. [14] conducted mechanical response tests on 12 Virginia Smart Roads. The tests consisted of transverse and longitudinal strains at the bottom of the asphalt layer and vertical pressure stresses at the surface, the bottom of the asphalt layer, and the top of the soil base. Bayat et al. [17] paved two asphalt pavement structures with a graded aggregate base and collected longitudinal tensile strains and compressive stresses. Ai et al. [2] measured the mechanical response within three pavement structures: semi-rigid base asphalt pavement, inverted pavement, and composite asphalt pavement. Cheng et al. [22,23,24] constructed and compared the mechanical response of a typical semi-rigid base asphalt pavement and a flexible base asphalt pavement under different temperature and speed conditions. The pavement structures for which mechanical response studies have been carried out can be divided into two main categories: asphalt pavements with flexible and semi-rigid bases. For flexible pavements, the base layer is mainly graded gravel or asphalt-treated crushed stone, whereas cement-stabilized crushed stone is mainly applied for semi-rigid base layers. Moreover, the buried sensors mainly include strain sensors, soil pressure cells, moisture sensors, and temperature sensors. The test protocol consists mainly of different vehicular speeds, tire pressures, and tire axle types. The above literature shows that various forms of asphalt pavements have been investigated, but there is a lack of research on applying fly ash and slag powder in large-size crushed stone base layers. In addition, the above studies’ load configuration, environment, and materials are relatively different from the pavement structures using a large-size crushed stone base treated with fly ash and slag powder. Therefore, it is necessary to further analyze the mechanical response of this type of asphalt pavement in conjunction with the physical engineering.

The objective of this paper is to investigate the mechanical response of asphalt pavements using a large-particle-size crushed stone base treated with fly ash and slag powder. Therefore, asphalt pavement was constructed using a large-particle-size crushed stone base treated with fly ash and slag powder. Asphalt pavement with a cement-stabilized crushed stone base was also constructed for comparison. Strain gauges and temperature sensors were installed at the bottom of the asphalt layer and at the bottom of the upper base layer. Then, the pavement strain response was collected at different dates after the pavement construction was completed. The effects of vehicular speed, base layer type, and measurement date on the strain response were compared. Analysis of variance (ANOVA) was carried out to investigate the impact of the interaction between factors. Finally, strain prediction equations were proposed. The research results can promote the application of large-particle-size crushed stone, fly ash, and slag powder in pavement engineering and provide a reference for pavement structure design using a large-particle-size crushed stone base treated with fly ash and slag powder.

## 2. Field Data Collection

### 2.1. Pavement Structures and Materials

**Figure 1:**
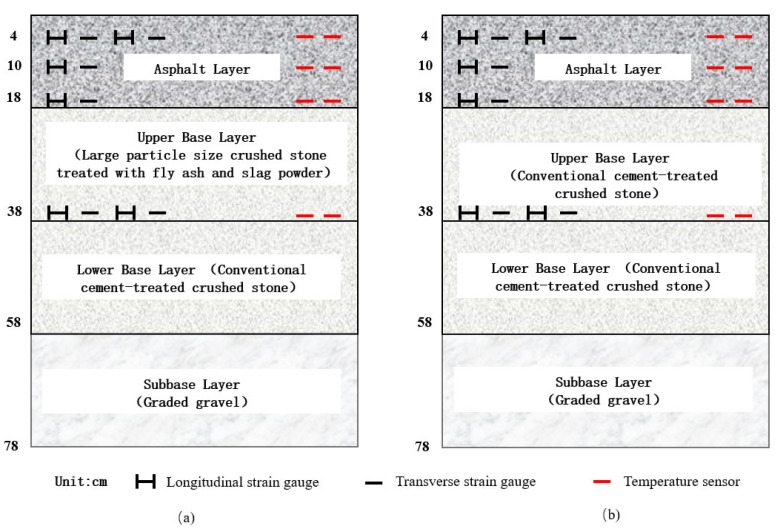
Pavement structure: (**a**) Section 1: the upper base layer used large-particle-size crushed stone treated with fly ash and slag powder; (**b**) Section 2: The upper base layer used cement-treated crushed stone.

**Figure 2:**
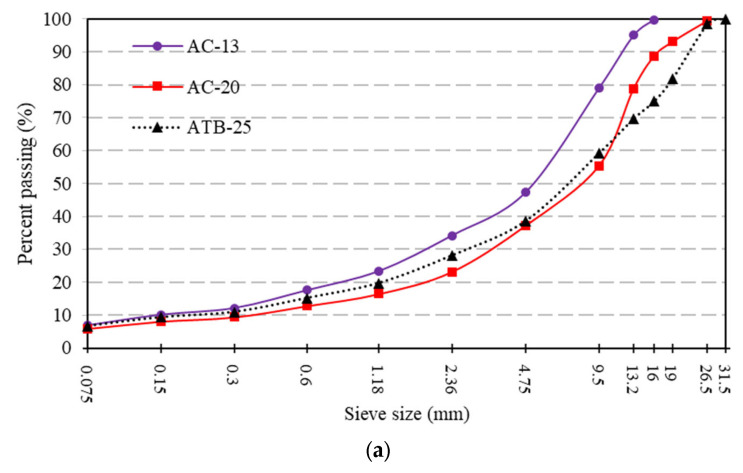

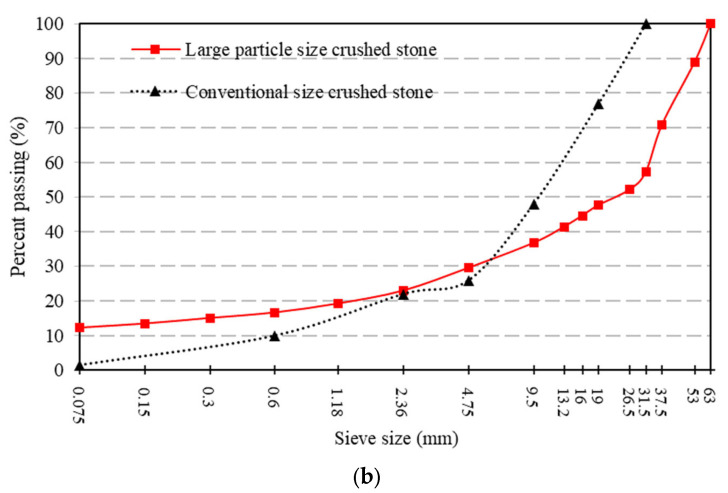
The aggregate gradation of (**a**) the asphalt layer and (**b**) the base layer.

The asphalt layer was divided into three layers: 4 cm AC-13, 6 cm AC-20, and 8 cm ATB-25. The AC-13 layer and the AC-20 layer used SBS-modified asphalt, and the ATB-25 layer used 70# base asphalt. The upper base was a large-particle-sized crushed stone base with fly ash and slag powder as the binder. The lower base was the most commonly used cement-stabilized gravel base in China. For comparison, cement-stabilized gravel material was also selected for the upper base. Details of the pavement structure are shown in Figure 1. Section 1 was used as an experimental section, and Section 2 was used for comparison. The aggregate gradation of the asphalt and base layers is shown in Figure 2.

As can be seen from Figure 1 and Figure 2b, the only difference between the experimental structure and the comparison structure was the material used in the upper base layer. For the experimental structure, fly ash and slag powder were used as binders, and cement material was used for the comparison structure. The slag powder used in this paper was granulated blast furnace slag powder. The specific surface area of the cement and the slag power was 328 m^2^/kg and 445 m^2^/kg, respectively. The fineness of the fly ash was 18.4%. In addition, aggregates with larger particle sizes were used in the experimental structure.

### 2.2. Strain Gauge Installation and Field Testing

#### 2.2.1. Installation of Strain Gauges

The strain gauge used in this paper was an “I”-type resistive strain gauge with a detection range of ± 1000 με, an operating temperature range of −20 °C~ + 200 °C, and a detection resolution of up to 0.1 με. Based on existing studies [1,24], the maximum value of the measured strain did not exceed 900 με. Therefore, the strain gauge used met the measurement requirements.

**Figure 3:**
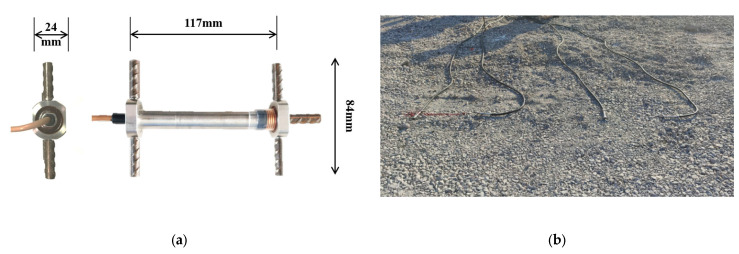

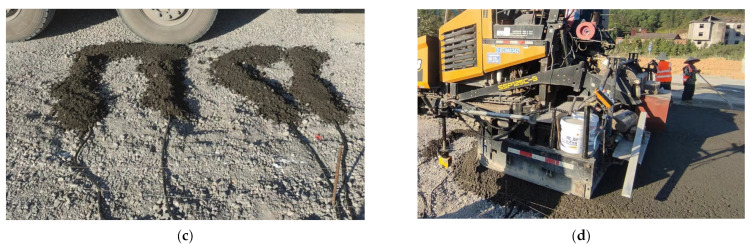
Strain gauge installation procedure at the bottom of the base layer: (**a**) strain gauge; (**b**) determination of the location of the strain gages and advance placement of the strain gages; (**c**) early burial of strain gauges; (**d**) base layer paving.

This means that it could meet the needs of the strain response detection of the asphalt layer and the base layer. The external dimension of the strain gauge was 24 m × 84 mm × 117 mm, as shown in Figure 3a.

During construction, strain gauges were buried in different layers of the asphalt layer and at the bottom of the upper base layer. The burial plan is shown in Figure 1. The embedding directions were horizontal and longitudinal, respectively. In addition, temperature sensors were simultaneously embedded to detect the temperature field inside the pavement. The strain gauges and on-site burial conditions are shown in Figure 3.

The strain gauges needed to be placed in a suitable location, as shown in Figure 3b. Pre-burial was performed when the paver was about to pave, as shown in Figure 3c. Finally, the paver was paved, and the strain gauges were buried inside the pavement. The process of embedding the temperature sensors was the same as that of the strain gauges.

#### 2.2.2. Strain Measurements

**Figure 4:**
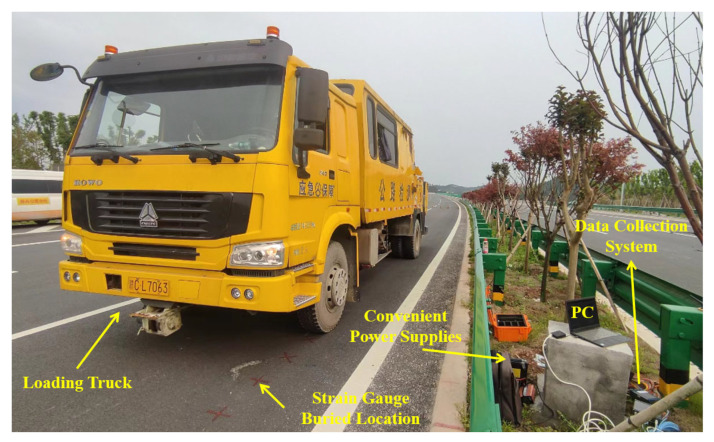
Field strain measurement.

This paper carried out vehicle loading on the structures where the strain gauges were embedded. The magnitude of the strain value was characterized using με. In other words, 1 με = 10^−6^ (m/m). A standard loading vehicle repeatedly drove to obtain the strain data, as shown in Figure 4. The rear axle weight of the vehicle was controlled to be 100 kN. At the same time, considering the influence of vehicle speed, the vehicle was driven repeatedly at 5 km/h, 10 km/h, 20 km/h, 40 km/h, and 60 km/h, with each speed condition being driven at least five times. In addition, this paper conducted tests on 12 October 2022, 12 April 2023, and 26 September 2023 to investigate the influence of temperature (service time) on the strain of asphalt pavement. When loading, one person was responsible for directing the vehicle to drive at the set speed and path to ensure that the tire was applied directly above the corresponding strain gauges. The other person collected data through the acquisition system. At the same time, the changes in the temperature field of the pavement during the measurement process were simultaneously recorded.

## 3. Results and Discussion

### 3.1. Strain Pulse Analysis

**Figure 5:**
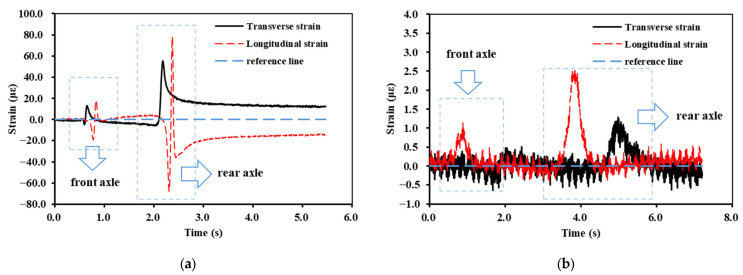
Typical transverse and longitudinal strain pulses: (**a**) the bottom of the AC-20 layer; (**b**) the bottom of the upper base layer.

The asphalt and base layer strain pulses were measured under vehicular loading. It was found that the strain pulses at different layers within the asphalt layer showed the same trend under three measurement time conditions. The strain pulse of the base layer was also the same. The typical pulses of longitudinal and transverse strains are shown in Figure 5.

Figure 5a shows the pulses of longitudinal and transverse strain measured in October 2022 for the bottom of the AC-20 layer applied at a 5 km/h vehicle speed. Figure 5b shows the strain pulse of the base layer measured on 12 October 2022. As depicted in Figure 5, the strain pulses of both the asphalt and the base layer coincided with the axle composition of the vehicle loading. Since the rear axle was larger than the front axle, the strain pulse of the rear axle was more significant than that of the front axle. The vehicle was kept at a constant speed during the strain measurement. Therefore, it can be assumed that the forces acting on the pavement surface by the rear axle during the loading process exhibited symmetrical distribution characteristics. From the pulses of the asphalt layer, it can be seen that both the longitudinal and the transverse strain showed prominent asymmetric characteristics. This phenomenon was due to the viscoelastic properties of the asphalt materials. The strain pulses of the base layer showed symmetrical characteristics, which indicates that the material used for the base layer was elastic. In addition, as presented in Figure 5, the strain inside the asphalt layer was much greater than in the base layer.

**Figure 6:**
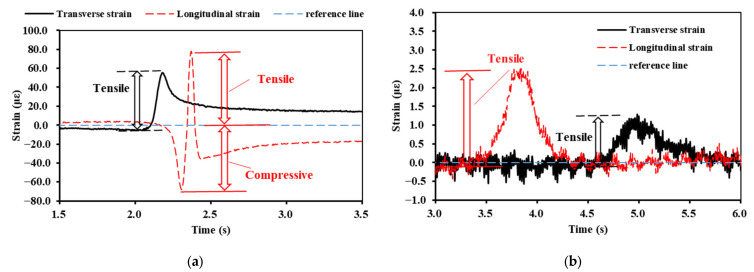
Strain pulses of the rear axle: (**a**) the bottom of the AC-20 layer; (**b**) the bottom of the upper base layer.

During the design of the asphalt pavement, the load was 100 kN. This type of load configuration is consistent with the axle weight of the rear axle of the vehicle loading used in this paper. Therefore, the strain pulse of the rear axle was further extracted, as shown in Figure 6.

As seen in Figure 6a, the transverse strain of the asphalt layer was mainly reflected as tensile strain, whereas the longitudinal strain showed alternating compressive–tensile–compressive strain. Furthermore, the reference line shows that neither the transverse nor the longitudinal strain fully recovered to the pre-loading state after the loaded vehicle had driven over the strain gauge. Figure 6b shows that, for the strain pulse of the base layer, both strain types were reflected as tensile strains. When the loaded vehicle drove over the strain gauge, its strain returned to its initial state.

### 3.2. Effect of Different Loading Conditions on Strain Response

The difference between the maximum and minimum values in the strain pulse was selected as a representative value. The representative values can reflect the influence of different loading conditions on the mechanical response of the pavement.

#### 3.2.1. Effect of Vehicular Speed on Representative Values of Strain

**Figure 7:**
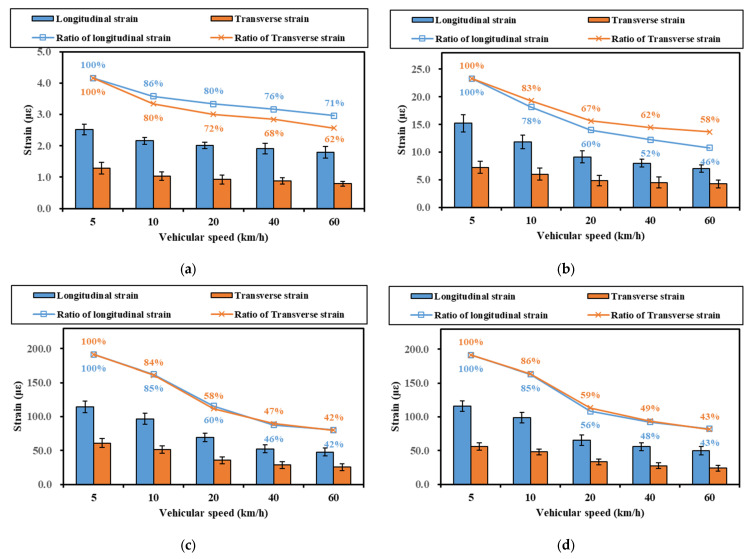
Effect of vehicular speed on representative values of strain in different layers: (**a**) strain at the bottom of upper base layer; (**b**) strain at the bottom of the ATB-25 layer; (**c**) strain at the bottom of the AC-20 layer; (**d**) strain at the bottom of the AC-13 layer.

The effect of different vehicular speeds on the representative values of strain was investigated first, as shown in Figure 7. The selected section was the experiment section (Section 1), and the measurement date was 12 October 2022. The material of the upper base layer in Section 1 used large-particle-size crushed stone treated with fly ash and slag powder, as depicted in Figure 1.

As shown in Figure 7, the strain representation values decreased with increasing speed for both the base and the asphalt layer. As the vehicular speed increased from 5 km/h to 60 km/h, the representative strain values decreased by 42% to 71%. For the strain at the bottom of the upper base layer, the decrease was relatively small compared to the asphalt layer, which implies that the asphalt layer was more sensitive to vehicular speed than the base layer. In addition, with the increase in speed, the decrease in the strain at the bottom of the ATB-25 layer was also smaller than that of the AC-20 layer and the AC-13 layer. It can be inferred that the effect of vehicular speed on strain decreased along the depth direction. Frequency is a factor that must be considered during pavement design. As we know, frequency positively correlates with the magnitude of speed [25], so careful consideration of vehicular speed is essential in the design process.

#### 3.2.2. Effect of Measurement Date on Representative Values of Strain

**Figure 8:**
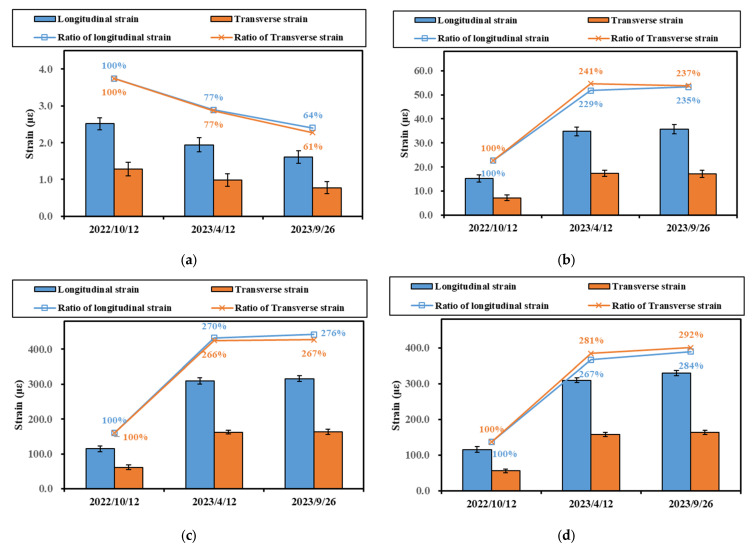
Effect of measurement date on representative values of strain in different layers: (**a**) strain at the bottom of the upper base layer; (**b**) strain at the bottom of the ATB-25 layer; (**c**) strain at the bottom of the AC-20 layer; (**d**) strain at the bottom of the AC-13 layer.

The strength of the base material grows over time. This change can significantly affect the mechanical responses within the pavement. Therefore, the effects of different measurement dates on pavement strain were investigated. Figure 8 illustrates the effect of different measurement dates on strain values. The measurement conditions are representative values of strain measured at 5 km/h for Section 1.

Figure 8 shows that the base layer’s strain value showed a decreasing trend with time, whereas the strain of the asphalt layer showed an increasing trend. For the representative strain value of the base layer, the strain decreased to 77% of the first measurement at the second measurement and to 61–64% at the third measurement. Since the base material is independent of temperature, it can be hypothesized that the base material’s stiffness became larger with service time, which led to a decrease in the value of the base strain. In addition, the decrease seen at the third measurement was significantly slower than that seen at the second measurement, and it can be inferred that the increase in base stiffness gradually slowed down.

For the asphalt layer, a significant increase in the representative values of the strains appeared at the second and third measurements compared to the first measurements. Based on the temperature sensor measurements, the temperatures in the middle of the asphalt layer at the three measurements were 27 °C, 34 °C, and 37 °C, respectively. Therefore, there should have been a significant difference between the second and third measurements of the asphalt layer. However, as shown in Figure 8, the difference between the second and third measurements was insignificant. This anomaly may be because the strength of the base layer was still increasing, leading to an increase in stiffness. The increase in stiffness and the increase in temperature showed an opposite trend, which led to a slight difference between the two measurements.

#### 3.2.3. Effect of Layer Positions on Representative Values of Strain

**Figure 9:**
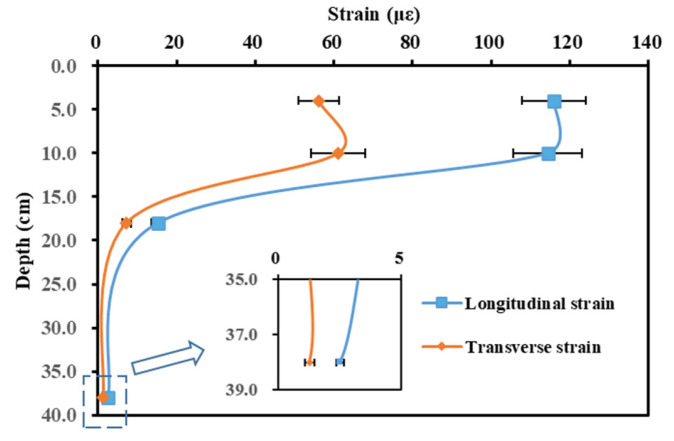
Effect of layer position on representative values of strain in different layers.

Further, the influence of layer positions on the representative values of strain was investigated. The results of the Section 1 measurements on 12 October 2022 were selected, and the measurement speed was taken as 5 km/h. The results are shown in Figure 9.

From Figure 9, it can be seen that the strain values showed an increasing and then decreasing trend with increasing depth. Compared to the 0–15 cm depth range, the 15–38 cm range strain values were minimal. Therefore, it can be assumed that the maximum strain value of the asphalt layer was located in the middle of the asphalt layer. In addition, in conjunction with Figure 7 and Figure 8, the longitudinal strain was about two times greater than the transverse strain.

#### 3.2.4. Effect of Base Layer Type on Representative Values of Strain

**Figure 10:**
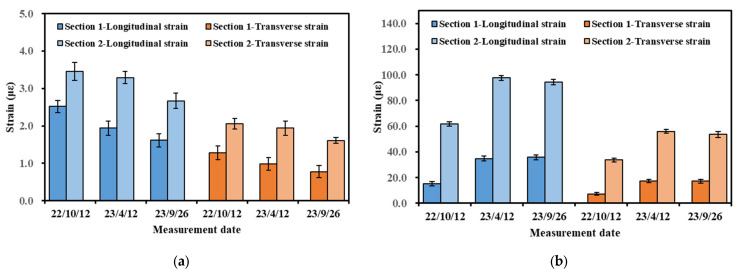

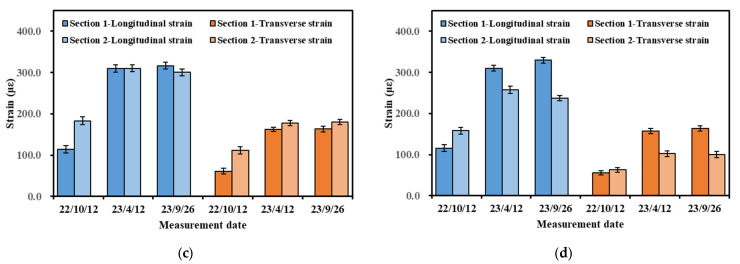
Effect of upper base layer type on representative values of strain in different layers: (**a**) strain at the bottom of the upper base layer; (**b**) strain at the bottom of the ATB-25 layer; (**c**) strain at the bottom of the AC-20 layer; (**d**) strain at the bottom of the AC-13 layer.

Finally, the effect of different upper base types on the representative values of strain was compared. The comparison results are shown in Figure 10. For the comparison, the data used were representative values of the strain obtained on 12 October 2022 while traveling at a 5 km/h speed. Section 1 treated the upper base layer with fly ash and slag powder using large-grained crushed stone. In contrast, Section 2 used the most commonly used cement-stabilized crushed stone. Except for the upper base layer, all other structural layer materials and construction processes were the same for Sections 1 and 2.

Figure 10 shows that, for the upper base layer and the ATB-25 layer, the strain value of Section 1 was smaller than that of Section 2. For asphalt layers, the lower the strain value, the less likely it is that cracking will occur. The result indicates that Section 1 was more advantageous than Section 2 for preventing bottom-up cracking. In other words, for Section 1, pavement cracking was more likely to occur in the upper and middle parts of the asphalt layer. In addition, for Section 1, the materials used were fly ash and slag powder, which also have a lower probability of reflective cracks than cement. Therefore, Section 1 had the characteristics of a long-life asphalt pavement during subsequent maintenance and repair. It can also be noted that the strain of Section 1 was higher than that of Section 2 at the shallower locations of the pavement at higher temperatures.

### 3.3. Statistical Analysis

The analysis in Section 3.2 indicated that vehicular speed, measurement date, and upper base type significantly affected the representative strain values. This section further explores the impact of the interaction of these factors on the strain values. Multifactor analysis of variance (three-way ANOVA) was utilized to analyze the effects of the interactions. Before analysis, the data were tested for normality using the Anderson–Darling (AD) test. This test defaults to data obeying a normal distribution, and if the *p*-value is less than 0.05, the original hypothesis is rejected and it is understood that the test data do not follow a normal distribution. Further, Bartlett’s method tested the data for homogeneity of variance. Bartlett’s test determines whether the variances between groups are equal by finding the chi-square statistic between the different groups. The *p*-value can also interpret the results of Bartlett’s method. When the *p*-value is greater than 0.05, the requirement of homogeneity of variance is satisfied.

The *p*-values for all data samples were greater than 0.05, which satisfied the requirements for performing three-way ANOVA. The results of the ANOVA for the longitudinal strain at the bottom of the AC-20 layer, for example, are shown in Table 1.

It can be seen from Table 1 that vehicular speed, measurement date, and base layer type had a significant impact on the representative values of longitudinal strain at the bottom of the AC-20 layer. Furthermore, the interaction between vehicular speed and measurement time was significant, whereas the interaction between vehicular speed and base layer type, base layer type and measurement date were insignificant. The different measurement dates represent different temperatures. Therefore, we can infer that the interaction of temperature and speed significantly impacted the representative strain values, which is consistent with the research results of Cheng et al. [26].

In addition, the results of transverse and longitudinal strain in other layers were statistically analyzed, as shown in Table 2.

As presented in Table 2, for the AC-13 layer and the AC-20 layer, the interactions between measurement date and base layer type were insignificant. The results may be related to the fact that the AC-13 layer and the AC-20 layer were far away from the base layer. Both vehicular speed and measurement time had significant effects on the representative strain values. Other positions significantly impacted the base layer type except for the transverse strain of the AC-13 layer and the upper base layer.

### 3.4. Strain Prediction Equations

Furthermore, based on the multifactor analysis results, models could be obtained for estimating the representative values of the strain of the asphalt layer and the base layer. For asphalt layers, the temperature is different for different measurement times. Since temperature significantly affects the strain in asphalt layers [26], a temperature metric was used here to quantify the effect of measurement time. For the base layer, the measurement time can be quantified by service time. The fitting equations used here are shown in Equations (1) and (2). Among them, Equation (1) was used to fit the representative strain value of the base layer, and Equation (2) was utilized to obtain the asphalt layer’s representative strain value.

(1) $$\varepsilon_{base-layer}=(a_{1}+b_{1}\cdot V)\cdot e^{c_{1}t}$$

(2) $$\varepsilon_{asphalt-layer}=(a_{2}+b_{2}\cdot V)\cdot e^{c_{2}T}$$

where *ε_base_* and *ε_asphalt-layer_* represent the representative strain values, με; *V* represents the vehicular speed, km/h; *t* represents the service time, year; *T* represents the temperature of the mid-depth of the asphalt layer, °C; and *a_1_*, *b_1_*, *c_1_*, *a*_2_, *b_2_*, and *c_2_* are the fitting parameters.

In developing Equations (1) and (2), the least square method was used, and the software used was the “fitting curve toolbox” in MATLAB. The fitting results of the base layer are shown in Table 3. The relevant results of the asphalt layer are shown in Table 4.

As depicted in Table 3 and Table 4, Equations (1) and (2) better fit the relationship between the representative value of the pavement and vehicular speed, temperature, or service time. Based on Table 3 and Table 4, the representative strain values of the pavement under different situations can be estimated, thereby providing a reference for similar pavement design and evaluation.

## 4. Conclusions

This research investigated the strain of pavement structures with a large-particle-size crushed stone base treated with fly ash and slag powder. Two different base types of asphalt pavement structures were paved. The strain response of the asphalt pavement was measured at different conditions. The impacts of different influencing factors on the strain values were examined using sensitivity analysis and multifactor ANOVA (analysis of variance), and strain prediction equations were developed. Based on the measured results, the following conclusions were summarized:

1. The transverse strain pulse of the asphalt layer was mainly characterized by tensile strain, whereas the longitudinal strain was characterized by alternating “tensile–compressive–tensile” Strain. For the base layer, both the transverse and the longitudinal strain were tensile. The type of base layer did not affect the strain pulse characteristics.
2. Based on the sensitivity analysis of the strain value, it can be seen that vehicular speed and measurement date had a relatively significant impact. The strain values of both the asphalt and the base layer decreased with increasing vehicular speed. In the case of the asphalt layers, the measurement date could be characterized using temperature values, and in the case of the base layers, using the service time indicator. Thus, the strain in the asphalt layer increased as the temperature increased, and the strain in the base layer decreased as the service time increased.
3. Compared to the conventional cement-stabilized crushed stone base, the base treated with fly ash and slag powder with large-particle-size crushed stone presented a smaller strain for the bottom of the upper base layer and the ATB-25 layer under different measurement dates. The results indicate that the upper base and the bottom of the asphalt layers were less prone to cracking, which could extend the life of the asphalt pavement.
4. The interaction of vehicular speed and temperature for the asphalt layers was significant. There was no interaction between vehicular speed, base type, and temperature, for the AC-20 and the AC-13 layers. Based on the results of multifactor analysis of variance, strain prediction equations for different base types with different positions were proposed. The prediction equations can provide a reference for pavement design and life prediction.

The article recommends using a large-particle-size crushed stone base treated with fly ash and slag powder, which reduces the generation of bottom-up cracks. However, only one year of strain data was measured in this paper. Future monitoring of the strain response within the pavement is needed to validate the results of this paper.

## Figures and Tables

**Table 1:** The results of three-way ANOVA (the longitudinal strain at the bottom of the AC-20 layer).

	Sum of Squares	DOF	Mean Square	F-Ratio	*p*-Value
Vehicular speed (X_1_)	52,380.3	4	13,095.1	121.728	0.000 *
Measurement date (X_2_)	17,784.7	2	8892.4	82.661	0.000 *
Base layer type (X_3_)	2933.6	1	2933.6	27.270	0.001 *
X_1_ × X_2_	8652.8	8	1081.6	10.054	0.002 *
X_1_ × X_3_	539.8	4	135.0	1.255	0.363
X_2_ × X_3_	78.2	2	39.1	0.364	0.706
Error	860.6	8	107.6		
Total	83,230.0	29			

* Statistically significant at 0.05.

**Table 2:** The result of three-way ANOVA (summary of results).

*p*-Value	Vehicular Speed (X_1_)	Measurement Date (X_2_)	Base Layer Type (X_3_)	X_1_ × X_2_	X_1_ × X_3_	X_2_ × X_3_
Longitudinal strain at the bottom of the upper base layer	0.000 *	0.000 *	0.001 *	0.049 *	0.000 *	0.001 *
Transverse strain at the bottom of the upper base layer	0.000 *	0.000 *	0.242	0.165	0.000 *	0.009 *
Longitudinal strain at the bottom of the ATB-25 layer	0.000 *	0.000 *	0.000 *	0.000 *	0.000 *	0.000 *
Transverse strain at the bottom of the ATB-25 layer	0.000 *	0.000 *	0.000 *	0.000 *	0.000 *	0.000 *
Longitudinal strain at the bottom of the AC-20 layer	0.000 *	0.000 *	0.001 *	0.002 *	0.363	0.706
Transverse strain at the bottom of the AC-20 layer	0.000 *	0.000 *	0.000 *	0.000 *	0.764	0.405
Longitudinal strain at the bottom of the AC-13 layer	0.000 *	0.000 *	0.000 *	0.000 *	0.240	0.112
Transverse strain at the bottom of the AC-13 layer	0.000 *	0.000 *	0.012 *	0.000 *	0.705	0.201

* Statistically significant at 0.05.

**Table 3:** The prediction equation of the base layer.

Section and Layer Positions		*a_1_*	*b_1_*	*c_1_*	R^2^
Section 1	Longitudinal strain at the bottom of the upper base layer	−0.0060	1.1553	−0.3993	0.88
	Transverse strain at the bottom of the upper base layer	−0.0097	2.3374	−0.3546	0.92
Section 2	Longitudinal strain at the bottom of the upper base layer	−0.0227	1.5619	−0.1512	0.62
	Transverse strain at the bottom of the upper base layer	−0.0294	2.7018	−0.1700	0.55

**Table 4:** The prediction equation of the asphalt layer.

Section and Layer Positions		*a_2_*	*b_2_*	*c_2_*	R^2^
Section 1	Longitudinal strain at the bottom of the ATB-25 layer	−0.0154	1.1284	0.0726	0.73
	Transverse strain at the bottom of the ATB-25 layer	−0.0275	1.9673	0.0771	0.76
	Longitudinal strain at the bottom of the AC-20 layer	−0.0824	5.7882	0.0905	0.80
	Transverse strain at the bottom of the AC-20 layer	−0.1548	10.9078	0.0907	0.80
	Longitudinal strain at the bottom of the AC-13 layer	−0.0718	5.0274	0.0932	0.79
	Transverse strain at the bottom of the AC-13 layer	−0.1430	10.0641	0.0934	0.80
Section 2	Longitudinal strain at the bottom of the ATB-25 layer	−0.0763	5.4749	0.0635	0.82
	Transverse strain at the bottom of the ATB-25 layer	−0.1448	10.3514	0.0612	0.81
	Longitudinal strain at the bottom of the AC-20 layer	−0.2345	16.9656	0.0654	0.83
	Transverse strain at the bottom of the AC-20 layer	−0.4147	29.9685	0.0644	0.81
	Longitudinal strain at the bottom of the AC-13 layer	−0.1329	9.5045	0.0652	0.82
	Transverse strain at the bottom of the AC-13 layer	−0.3530	25.5242	0.0630	0.81

## Data Availability

The data presented in this study are available on request from the corresponding author.
